# Supplementary material for: In situ architecture of the Tad pilus machine in Caulobacter crescentus
Source: mBio. 2026 Mar 25;17(5):e00111-26. doi: 10.1128/mbio.00111-26 (PMC13170226; doi:10.1128/mbio.00111-26)
Supplement: Supplemental Material — Supplemental figures, tables, and movie legends. [file mbio.00111-26-s0001.pdf]

**SUPPLEMENTARY INFORMATION**

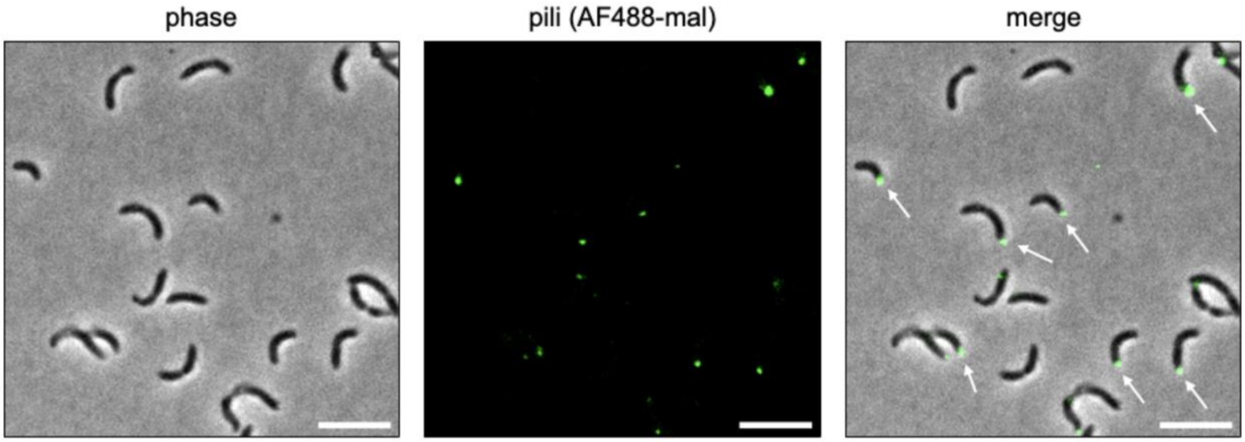


**Figure S1. The *Cc*60L mutant of *C. crescentus* produces pili that are immobilized during the early stages of extension.** Representative microscopy images of mixed populations of the *C. crescentus Cc*60L mutant (bNY30a Δ*hfsDAB pilA2*$^{T36C,\ stop60L}$) labeled with AF488-maleimide (AF488-mal, green), which reacts with the engineered cysteine residue in the major pilin, PilA. Scale bars, 5 µm. White arrows indicate cells with a single polar fluorescent focus, corresponding to the production of very short pili.

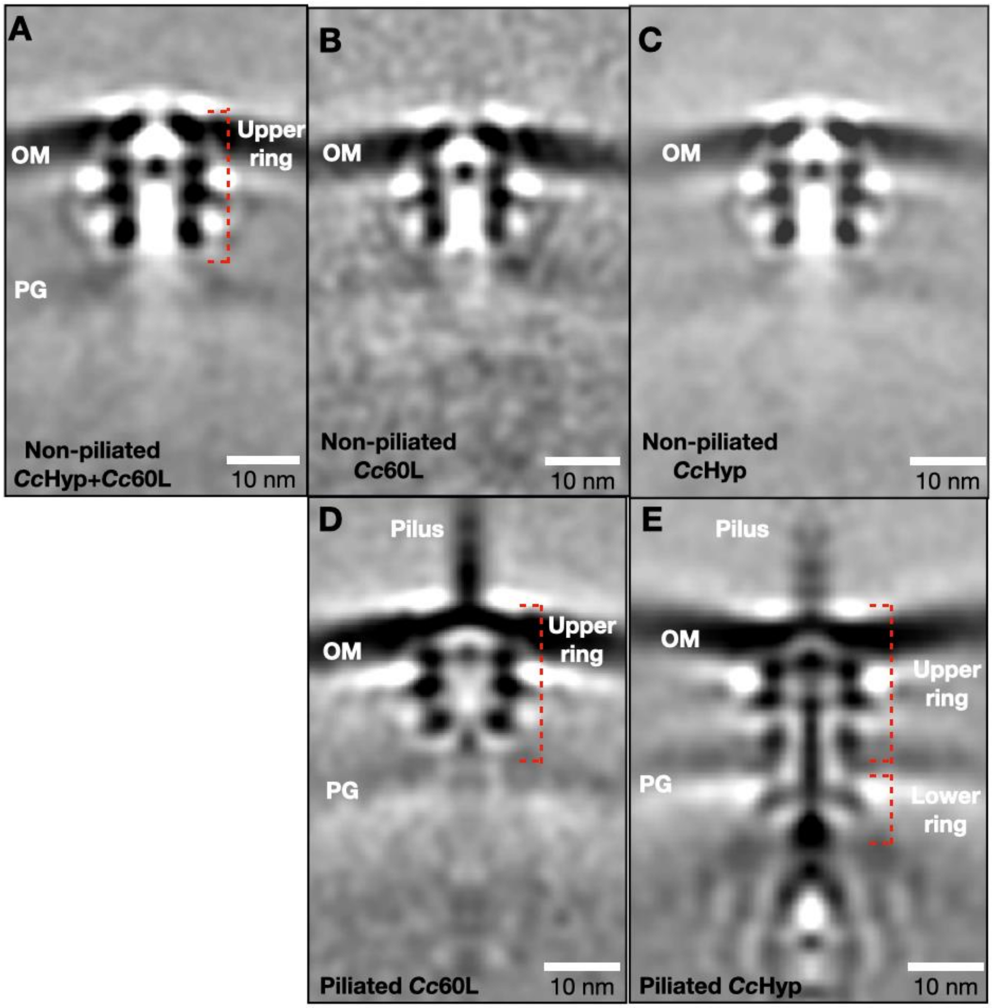


**Figure S2. Initial subtomogram averages of *Cc*Tad reveal a prominent OM ring-like density. A:** A central slice of the initial subtomogram-averaged structure of the non-piliated *C. crescentus Cc*Tad machines reveal an OM associated ring-like density. PG: peptidoglycan. **B-C:** Central slices of the subtomogram-averaged structures of the non-piliated *Cc*60L and *Cc*Hyp machines, respectively. **D:** A central slice of the subtomogram-averaged structure of the piliated *Cc*60L machines reveal only the OM-associated ring-like density despite extended pilus. **E:** A central slice of the subtomogram-averaged structure of the piliated *Cc*Hyp machines.

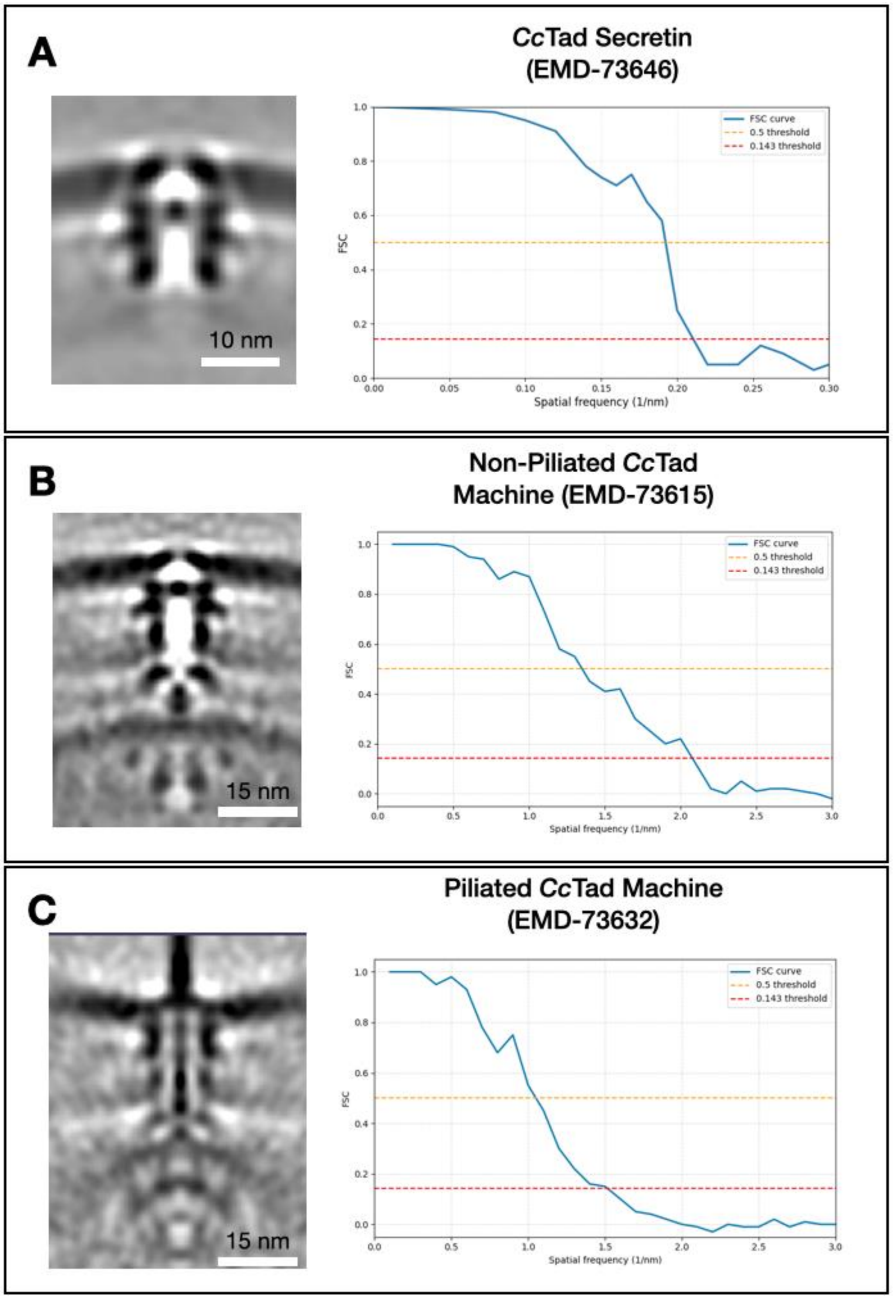


**Figure S3: Fourier shell correlation (FSC) curves for subtomogram averaged *Cc*Tad structures. A-C:** FSC curves used to estimate the resolution of the subtomogram averaged structures of (A) the *Cc*Tad secretin (38 Å, EMD-73646), (B) the non-piliated *Cc*Tad machine (42 Å, EMDB-73615) and (C) the piliated *Cc*Tad machine (56 Å, EMDB-73632).

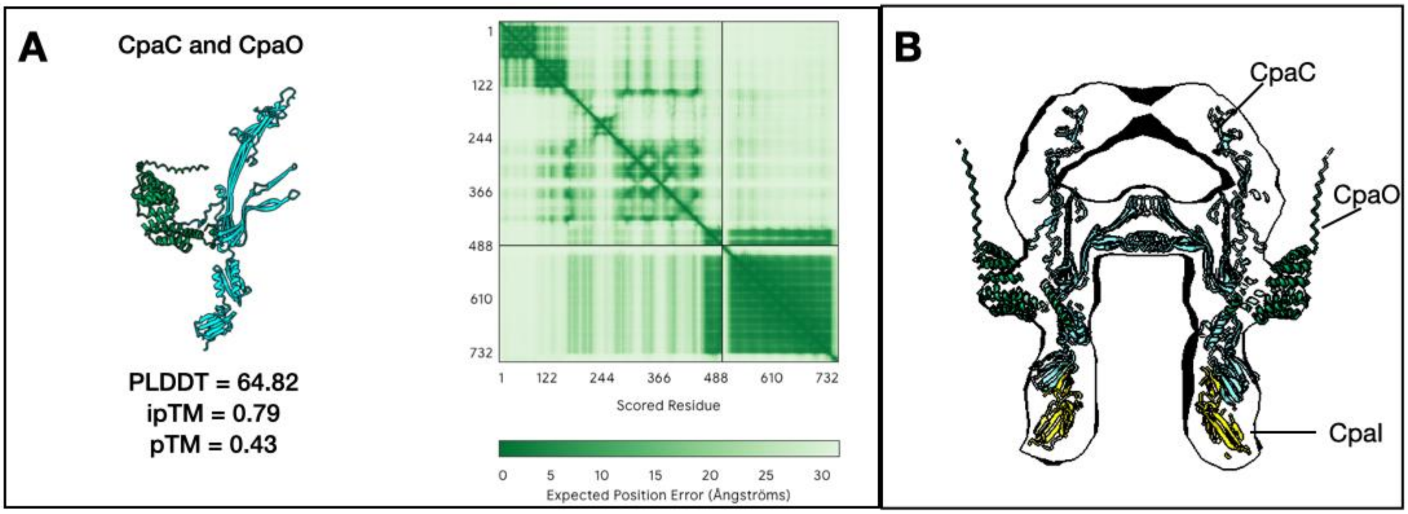


**Figure S4. AlphaFold3-predicted structure of CpaO fitted poorly to the spoke-like density within the outer membrane. A:** AlphaFold3 multimer prediction of CpaO in complex with CpaC, with associated confidence metrics PLDDT, ipTM and pTM. **B:** AlphaFold3 model of CpaO fit into the subtomogram-averaged map from Fig. 3A.

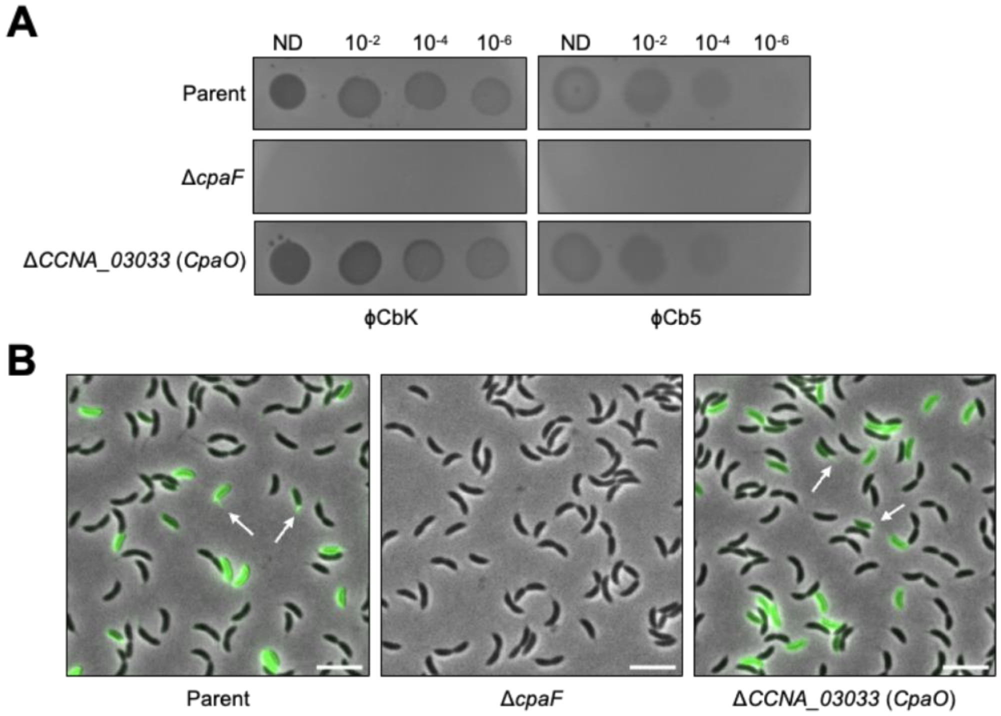


**Figure S5. The predicted TadD ortholog CCNA_03033 (CpaO) is not required for pilus production in *C. crescentus*. A:** Susceptibility of *C. crescentus* strains to the pilus-dependent phages ΦCbK and ΦCb5. Serial dilutions of phage are spotted onto bacterial lawns of the indicated strains incorporated into top agar. The presence of plaques is indicative of phage-mediated bacterial lysis, which is dependent on the elaboration of a pilus. The parental strain is NA1000 *pilA*$^{T36C}$, into which Δ*cpaF* and Δ*CCNA_03033* mutations were introduced to generate the respective mutant strains. ND, no dilution. **B:** Representative microscopy images of mixed populations of the indicated *C. crescentus* strains labeled with AF488-maleimide (green), which reacts with the engineered cysteine residue in the major pilin, PilA. Cell body fluorescence results from the retraction of extracellularly labeled pilus filaments into the cell. Scale bars, 5 µm. White arrows indicate cells with pili in the extended state.

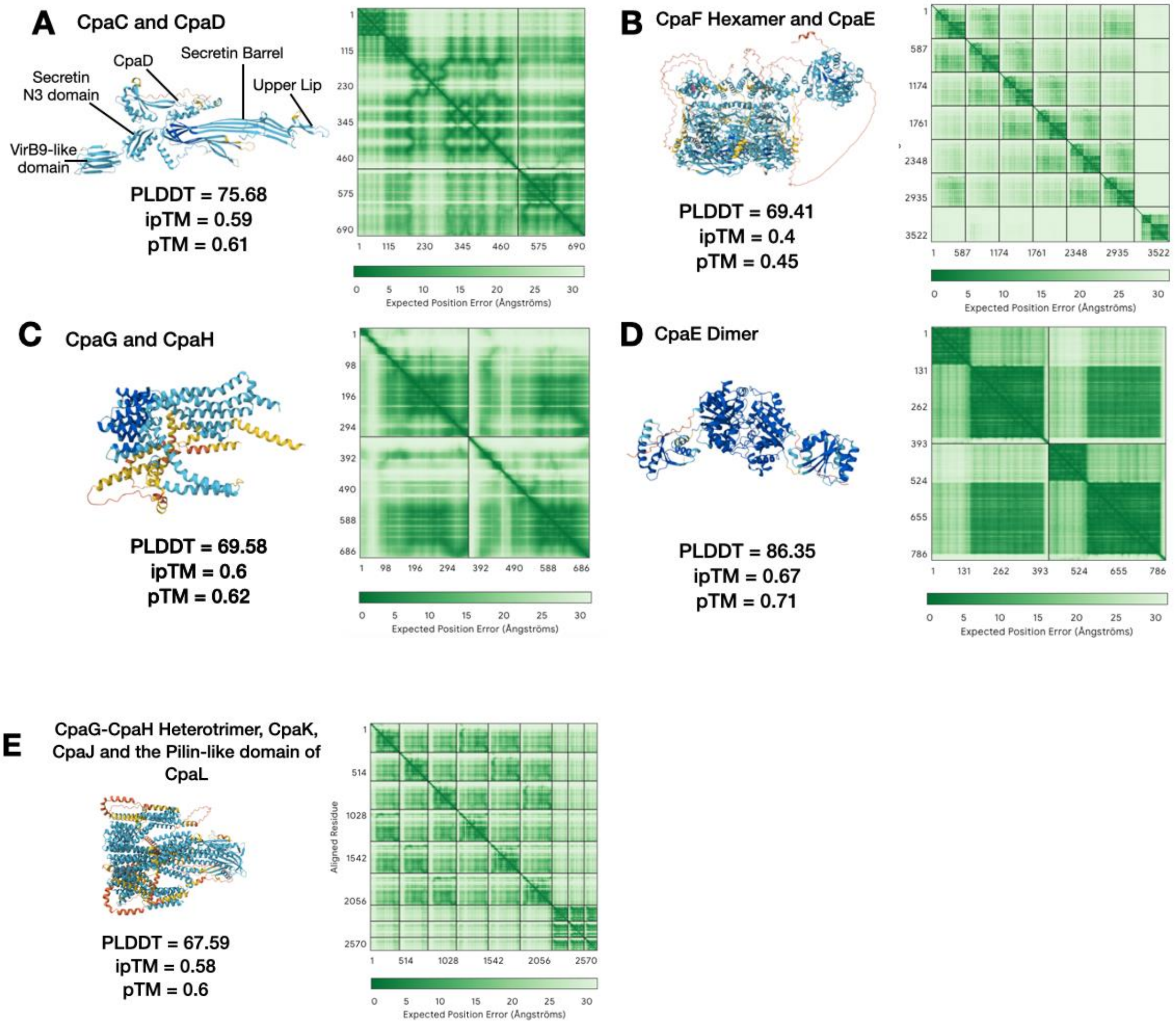


**Figure S6. AlphaFold3-predicted models and statistics of the tad pilus machine components.** Modelling statistics are shown for the highest-ranked AlphaFold3 models. **A.** AlphaFold3-predicted model of interactions between monomeric CpaC and CpaD. **B.** AlphaFold3-predicted model of interactions between hexameric CpaF and monomeric CpaE. **C.** AlphaFold3-predicted model of interactions in a heterodimer of CpaG and CpaH. **D.** AlphaFold3-predicted model of a homodimer of CpaE. **E**. AlphaFold3-predicted model of a multimer of monomeric CpaK, CpaJ and the pilin-like domain of CpaL, with a trimer of CpaG/CpaH heterodimers.

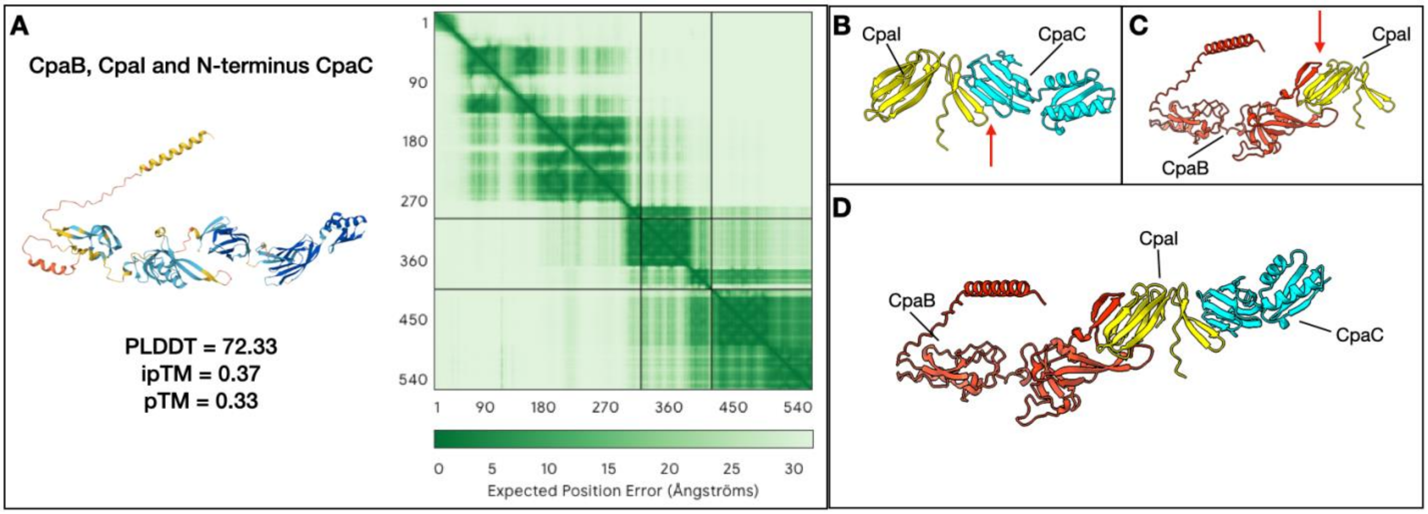


**Figure S7. AlphaFold3-predicted CpaI interacts with CpaB and CpaC via donor-strand complementation-like interactions. A:** Modelling statistics PLDDT, ipTM and pTM as returned by AlphaFold3 for the complex of CpaB, CpaI and the N-terminal domain of CpaC. **B.** AlphaFold3 model from (A) visualized to highlight the binding interface between CpaI and CpaC, indicated by a red arrow. **C:** AlphaFold3 model from (A) visualized to highlight the binding interface between CpaI and CpaB, indicated by a red arrow. **D:** AlphaFold3 model from (A) with each component coloured individually.

**Table S1. Bacterial strains, plasmids, and primers used in this study.**

| Strain | Description | Source |
|---|---|---|
| ***Escherichia coli* strains** | | |
| DH5α | Cloning strain; F⁻ *mcrA* Δ(*mrr-hsd*RMS-*mcr*BC) φ80*lacZ*ΔM15 Δ*lac*X74 *rec*A1 *ara*D139 Δ(*ara-leu*)7697 *gal*U *gal*K λ–*rps*L(Str$^R$) *end*A1 *nup*G | Invitrogen |
| NEB5α | DH5α derivative, *fhuA2Δ*(*argF-lacZ*)*U169 phoA glnV44 Φ80Δ*(*lacZ*)*M15 gyrA96 recA1 relA1 endA1 thi-1 hsdR17* | New England Biolabs |
| YB10335 | NEB5α pNPTS138::Δ*CCNA_03033* | This study |
| YB10336 | NEB5α pNPTS138::*pilA2*$_{\text{CB13}}^{\text{T36C, stop60L}}$ | This study |
| ***Caulobacter crescentus* strains** | | |
| NA1000 | Synchronizable *C. crescentus* lab adapted strain that does not produce a holdfast | Evinger and Agabian, 1977 |
| bNY30a | Hyperpiliated derivative of CB13b1a, originally SW16-Pil200 | Lagenaur and Agabian, 1977 |
| YB8288 | NA1000 *pilA*$^{\text{T36C}}$; pili can be labelled with maleimide-conjugated fluorophores | Ellison et al. 2017 |
| YB8446 | NA1000 *pilA*$^{\text{T36C}}$ Δ*cpaF*, unmarked, non-polar deletion of the *cpaF* ORF | Yen et al. 2025 |
| YB10337 | NA1000 *pilA*$^{\text{T36C}}$ Δ*CCNA_03033*, allelic exchange with plasmid from YB10335 electroporated into YB8288 | This study |
| YB9034 | bNY30a Δ*hfsDAB pilA2*$^{\text{T36C}}$; pili can be labelled with maleimide-conjugated fluorophores, does not produce holdfast | Ellison et al. 2019 |
| YB10338 | bNY30a Δ*hfsDAB pilA2*$^{\text{T36C, stop60L}}$; allelic exchange with plasmid from YB10336 electroporated into YB9034 | This study |
| **Plasmid** | | |
| pNPTS138 | Litmus 38 derivative, *nptI oriT sacB, Kan*$^R$; used for allelic exchange in *C. crescentus* | M.R.K Alley, unpublished |
| pNPTS138:: Δ*CCNA_03033* | pNPTS138 containing 501 bp upstream of *CCNA_03033* codon 13 fused to 499 bp downstream of *CCNA_03033* codon 259 at the EcoRV site; used to generate an in-frame, markerless deletion of the *CCNA_03033* ORF from the NA1000 genome | This study |

| | | |
|---|---|---|
| pNPTS138:: *pilA2*$_{\mathrm{CB13}}^{\mathrm{T36C,\ stop60L}}$ | pNPTS138 containing a genomic fragment 298 bp upstream of the *pilA2* start codon to 442 bp downstream of the *pilA2* stop codon at the EcoRV site, contains the T36C and stop60L point mutations in the *pilA2* ORF, used to introduce the stop60L mutation into the *pilA2* ORF from the bNY30a genome | This study |
| **Primer** | | |
| Δ*CCNA_03033*-upF | **GCCAAGCTTCTCTGCAGGAT**TCGGCTCCCCGCGCTTCT | This study |
| Δ*CCNA_03033*-upR | *CCAGCTGCGGGACTGACC*GAGAACGGTTGCGATGAGCG | This study |
| Δ*CCNA_03033*-downF | GGTCAGTCCCGCAGCTGG | This study |
| Δ*CCNA_03033*-downR | **GCGAATTCGTGGATCCAGAT**TGCGGGTCATGGCCAAGGAA | This study |
| *pilA2*$^{\mathrm{T36C\ stop60L}}$-upF | **GCCAAGCTTCTCTGCAGGAT**TCCGAACAGCCCCCAAGATCACTTT | This study |
| *pilA2*$^{\mathrm{T36C\ stop60L}}$-upR | *CGCGGGTGGCTaAGGTGCCAGCC*GCCGTCGACACCGCCGTACC | This study |
| *pilA2*$^{\mathrm{T36C\ stop60L}}$-downF | *GGCTGGCACCTtAGCCACCCGCG*CCACGCTCGAAAAGAAGAGGGTCGA | This study |
| *pilA2*$^{\mathrm{T36C\ stop60L}}$-downR | **GCGAATTCGTGGATCCAGAT**CAAAGAAAAGGGCCGAGGCGGTTAG | This study |

*Sequences for Gibson assembly into destination plasmids are bolded; regions of complementarity to the target amplicon are underlined; regions of reverse complementarity to facilitate splicing are italicized; nucleotides that differ from the coding sequence (to introduce point mutations) are indicated in lower case.

## Description of Additional Supplementary Files

**Supplementary Movie S1:** Time-lapse of *Cc*Hyp (bNY30a Δ*hfsDAB pilA2*$^{\mathrm{T36C}}$) cells extending and retracting pili after labeling with AF488-maleimide (green). Capture rate is 3 sec/frame. Scale bars, 5 μm.

**Supplementary Movie S2:** Schematic overview of the process of *in situ* cryo-electron Tomography on a *Cc*Hyp cell (bNY30a Δ*hfsDAB pilA2*$^{T36C}$), illustrating the resolved architecture of the Tad pilus machine in *Caulobacter crescentus*.
